# Novel Large Sulfur Bacteria in the Metagenomes of Groundwater-Fed Chemosynthetic Microbial Mats in the Lake Huron Basin

**DOI:** 10.3389/fmicb.2017.00791

**Published:** 2017-05-08

**Authors:** Allison M. Sharrar, Beverly E. Flood, Jake V. Bailey, Daniel S. Jones, Bopaiah A. Biddanda, Steven A. Ruberg, Daniel N. Marcus, Gregory J. Dick

**Affiliations:** ^1^Department of Earth and Environmental Sciences, University of Michigan, Ann ArborMI, USA; ^2^Department of Earth Sciences, University of Minnesota, MinneapolisMN, USA; ^3^BioTechnology Institute, University of Minnesota, MinneapolisMN, USA; ^4^Annis Water Resources Institute, Grand Valley State University, MuskegonMI, USA; ^5^NOAA-Great Lakes Environmental Research Laboratory, Ann ArborMI, USA

**Keywords:** chemosynthesis, dissimilatory sulfate reduction, sulfur oxidation, archaea, archaeal genomics, microbial mat, *Beggiatoa*, *Thiothrix*

## Abstract

Little is known about large sulfur bacteria (LSB) that inhabit sulfidic groundwater seeps in large lakes. To examine how geochemically relevant microbial metabolisms are partitioned among community members, we conducted metagenomic analysis of a chemosynthetic microbial mat in the Isolated Sinkhole, which is in a deep, aphotic environment of Lake Huron. For comparison, we also analyzed a white mat in an artesian fountain that is fed by groundwater similar to Isolated Sinkhole, but that sits in shallow water and is exposed to sunlight. *De novo* assembly and binning of metagenomic data from these two communities yielded near complete genomes and revealed representatives of two families of LSB. The Isolated Sinkhole community was dominated by novel members of the *Beggiatoaceae* that are phylogenetically intermediate between known freshwater and marine groups. Several of these *Beggiatoaceae* had 16S rRNA genes that contained introns previously observed only in marine taxa. The Alpena fountain was dominated by populations closely related to *Thiothrix lacustris* and an SM1 euryarchaeon known to live symbiotically with *Thiothrix* spp. The SM1 genomic bin contained evidence of H_2_-based lithoautotrophy. Genomic bins of both the *Thiothrix* and *Beggiatoaceae* contained genes for sulfur oxidation via the rDsr pathway, H_2_ oxidation via Ni-Fe hydrogenases, and the use of O_2_ and nitrate as electron acceptors. Mats at both sites also contained Deltaproteobacteria with genes for dissimilatory sulfate reduction (*sat, apr*, and *dsr*) and hydrogen oxidation (Ni-Fe hydrogenases). Overall, the microbial mats at the two sites held low-diversity microbial communities, displayed evidence of coupled sulfur cycling, and did not differ largely in their metabolic potentials, despite the environmental differences. These results show that groundwater-fed communities in an artesian fountain and in submerged sinkholes of Lake Huron are a rich source of novel LSB, associated heterotrophic and sulfate-reducing bacteria, and archaea.

## Introduction

The discovery of chemosynthetic communities at deep-sea hydrothermal vents fundamentally changed views of how energy and carbon flows through ecosystems (Jannasch and Wirsen, 1979). Although this marked the first known example of complex communities based on inorganic energy sources, the concept of chemosynthesis dates back far earlier, to Winogradsky’s studies of large sulfur bacteria (LSB) (Ackert, 2006). Chemosynthetic microbial communities have now been well studied in many marine and terrestrial environments. Particularly prominent are LSB that form dense mats at redox boundaries where sulfide meets oxygen (Jørgensen, 2010). These organisms are geographically widespread, feature extensive morphological diversity, and provide a rich platform for studying microbial evolution and physiology (Salman et al., 2013). LSB thrive in marine seeps (Larkin et al., 1994; Joye et al., 2004; Jones et al., 2015), mud volcanoes (Girnth et al., 2011), deep-sea hydrothermal systems (McKay et al., 2012), organic-rich sediments, terrestrial hydrothermal vents (Konkol et al., 2010) and sulfide springs (Caldwell et al., 1975; Nelson and Castenholz, 1981; Larkin et al., 1994; Teske, 2006; Chaudhary et al., 2009), sulfidic caves (Engel et al., 2001; Macalady et al., 2006) and phototrophic mats (Hinck et al., 2011). Although *Thioploca* spp. have been studied in some large lakes (Zemskaya et al., 2009), little is known about chemosynthetic communities or LSB in large freshwater lakes such as the Laurentian Great Lakes (Biddanda et al., 2006).

In submerged sinkholes of Lake Huron, venting of low-oxygen, saline, sulfate-rich, sulfidic groundwater supports chemosynthetic microbial communities (Biddanda et al., 2006). The geochemistry of this groundwater is shaped by water-rock interactions in Silurian-Devonian carbonate-limestone bedrock aquifers, in which evaporites including gypsum and anhydrite are dissolved (Ruberg et al., 2008). Dissolution of the limestone has produced both onshore and offshore karst formations in Alpena County, including sinkholes in the lake bottom where groundwater emerges (Biddanda et al., 2006). The sinkholes were first scientifically discovered during a 2001 shipwreck survey in the Thunder Bay National Marine Sanctuary (Coleman, 2002). During subsequent surveys in 2002 and 2003, active groundwater intrusion was observed at the Middle Island, Isolated, and Misery Bay sinkholes (Biddanda et al., 2006).

Several microbial mat communities that are not typically found elsewhere in the Great Lakes populate these sinkholes. At the Middle Island Sinkhole, mat communities in the photic zone at 23 m water depth are dominated by metabolically versatile cyanobacteria that can conduct both oxygenic and anoxygenic photosynthesis (Nold et al., 2010; Voorhies et al., 2012). In communities beneath the cyanobacterial mats chemosynthetic primary production is fueled by redox reactions of an active sulfur cycle driven by sulfur-oxidizing and sulfate-reducing bacteria (Nold et al., 2010; Voorhies et al., 2012).

The Isolated Sinkhole sits in the aphotic zone at a water depth of 93 m and covers an area of approximately 55 m × 40 m, as described in detail in Biddanda et al. (2006). Groundwater seeps into the sinkhole and is characterized by high chlorine, sulfate, phosphorus, particulate organic matter, and conductivity. Dissolved oxygen levels are low, ranging from between 1.28 and 5.12 mg L^-1^ throughout the year. A 1–2 m thick turbid “sinkhole plume” with elevated biomass sits near the bottom of the sinkhole. Two distinct microbial mat morphotypes cover the floor of the sinkhole: one is white, irregularly shaped, and cohesive and the other is brown, non-cohesive, and sometimes associated with pink ‘fingers’ (Biddanda et al., 2006). Previous sampling and microscopic analysis of the Isolated Sinkhole characterized the microbial mat community as low diversity, with just a few dominant bacterial species (Biddanda et al., 2006). Evidence of chemoautotrophic primary production at this site led to the conclusion that the microbial community generates most of its organic matter *in situ* rather than relying on organic material settling down from above. This study also predicted that dissimilatory sulfate reduction would be important in the sinkhole ecosystem, but the specific metabolisms and organisms have, until now, not been confirmed.

Groundwater seeps in this area also occur in sinkholes on land and at the Alpena County Library fountain, where an artesian well draws groundwater from the same aquifers that feed the sinkholes (Snider et al., 2017). The fountain consists of a lower basin that has brown cyanobacterial mats and an upper basin that has white chemosynthetic mats. A complete description of the fountain site is provided in Snider et al. (2017). Major differences in microbial mat habitat between this site and the Isolated Sinkhole include the following: (i) the fountain is exposed to direct sunlight, (ii) groundwater at the fountain is in closer contact with atmospheric oxygen but does not interact with lake water as at Isolated Sinkhole, (iii) mats in the fountain have no underlying sediment as at Isolated Sinkhole, (iv) the rate of water flow at the fountain is higher than at the Isolated Sinkhole.

In this study, we sought to understand the interaction between groundwater geochemistry and microbial community composition and metabolism in two contrasting settings, the Isolated Sinkhole and the Alpena fountain. We used a metagenomic approach to identify genes that underpin energy metabolisms, focusing on sulfur metabolism, hydrogen oxidation, aerobic respiration, denitrification, and carbon fixation. Our results indicate that these two communities are driven by similar suites of largely sulfur-based metabolic pathways held within different taxa at the two sites.

## Materials and Methods

### Sample Collection, Chemical Analyses, DNA Extraction, and Metagenomic Sequencing

A sample was collected from the whole white mat, which was directly attached to the fountain substrate, in the upper basin of the Alpena library fountain site (45 3.747′N, 83 25.872′W) on July 24th, 2012, and preserved in RNAlater (Ambion) as instructed by the manufacturer. Sulfide concentrations were determined by the methylene blue method (Cline, 1969) using Procedure 1 described by Reese et al. (2011). Dissolved oxygen was measured by a YSI Pro Dissolved Oxygen probe (Digital Professional Series). At the Isolated Sinkhole site (45 10.711′N, 83 09.173′W), a sample of white mat was skimmed off of sediment from a box core collected on September 6th 2008 on board the *R/V Laurentian* using a 60 ml syringe with attached flexible Tygon tubing, and immediately frozen at -20°C. After thawing, the white mat was manually separated from surrounding sediment and washed gently with a filter-sterilized NaCl solution to remove visible sediment particles. We note that even such gentle washing could reduce the diversity detected, and since it was only applied to the Isolated Sinkhole sample (where attached sediments were present), comparisons between the two samples could theoretically have been affected. However, it is unlikely to influence the results of our main focus, the large sulfur-oxidizing bacteria. DNA from both samples was extracted using the FastDNA Spin Kit for Soil (MP Biomedicals). Both samples were then sequenced in September of 2012 by standard methods using the Illumina HiSeq with paired-end 100 base-length reads at the University of Michigan DNA Sequencing Core. Sequence reads were deposited to the NCBI Sequence Read Archive (see **Supplementary Table S1** for accession numbers).

### rRNA Gene Assembly and Phylogenetic Analysis

16S rRNA gene sequences were assembled from the metagenomic datasets using EMIRGE (Miller et al., 2011). EMIRGE assemblies were performed with 120 iterations against the Silva small subunit reference database v. 111 (Pruesse et al., 2007) using quality filtered and trimmed reads as described below. Nine of the resulting 22 EMIRGE sequences had top BLAST hits within the *Beggiatoaceae*. These sequences were screened for chimeric sequences using Pintail (Ashelford et al., 2005) in mothur v. 1.33 (Schloss et al., 2009), and no chimeras were detected. Initial difficulties in resolving the phylogeny of one the EMIRGE sequences suggested the possible presence of an intron. The EMIRGE sequences were aligned with 16S rRNA gene sequences from taxa within the *Beggiatoaceae* that contain introns, as well as taxa whose 16S rRNA gene sequences do not contain introns, using Geneious (9.1.3) (Kearse et al., 2012). This alignment identified homologs to introns observed by Salman et al. (2012). The introns were manually removed and the 16S rRNA genes were aligned using the SINA aligner (Pruesse et al., 2012). The alignment was manually corrected in ARB (Ludwig et al., 2004) before phylogenetic analyses. Alignments were end-clipped so that all sequences were of equal length and positions with >50% gaps were removed (final alignment 28- 1271 *E. coli* base positions). Tree building was performed within Geneious. A neighbor joining phylogenetic tree with Jukes-Cantor distance correction (Jukes and Cantor, 1969; Saitou and Nei, 1987) and a heuristic search maximum parsimony tree (Fitch, 1971) were constructed with PAUP 4.0a148 (Swofford, 2003). Both trees were bootstrapped (Felsenstein, 1985) with 1000 replicates and consensus trees were generated. A consensus Bayesian interference phylogenic tree was constructed with MrBayes 3.2.6 with 1,000,000 generational runs (Huelsenbeck and Ronquist, 2001) using six substitution categories (aka. the general time reversible (GTR) model), gamma distribution and an invariant site nucleotide substitution rate (G++I). The trees were saved every 100 generations and posterior probabilities calculated after discarding the first 20% of trees. For maximum likelihood analyses, the GTR nucleotide substitution model with G+I rate variation was selected with JModelTest v.2.1.10 (Darriba et al., 2012). The Maximum-Likelihood (ML) analysis was computed in RAxML v.7.2.8 (Stamatakis, 2006) with 1000 bootstrap replicates. The ML tree was selected for the phylogenic distance tree. Branches were collapsed when ML bootstrap values fell below 50%.

### Phylogenetic Analysis of rpoB

*rpoB* phylogenies were calculated using full-length or nearly full-length *rpoB* gene sequences from the metagenomes, and from freshwater and marine *Beggiatoaceae* available from isolate genomes and metagenomes at the Joint Genome Institute Integrated Microbial Genomes and Metagenomics (IMG/M) website (Markowitz et al., 2008). Nucleotide sequences were translated in ARB (Ludwig et al., 2004). Protein sequences were aligned using M-Coffee (Moretti et al., 2007), re-imported into ARB, and the corresponding nucleotide sequences re-aligned according to the amino acid alignments. Additional sequences were aligned with the ARB integrated aligner. Maximum likelihood analyses were computed using the rapid bootstrap algorithm (1000 replicates) in RAxML v.8.0.24 (Stamatakis, 2006). All positions with missing information and gaps were masked from the alignments prior to analysis, for final lengths of 989 and 2967 columns for the protein and nucleotide alignments, respectively. Evolutionary models were selected using the corrected Akaike information criterion (AICc) with ProtTest v.2.4 (Abascal et al., 2005) and JModelTest v2.1.5 (Darriba et al., 2012). Maximum likelihood analyses of protein sequences were calculated with the LG amino acid replacement matrix (Le and Gascuel, 2008) and gamma-distributed rates, with proportions of invariant sites and the alpha parameter estimated from the data. Maximum likelihood analyses of nucleotide sequences were calculated with the general time reversible model, gamma-distributed rates, and proportions of invariant sites and the alpha parameter estimated from the data.

### Whole Genome Assembly and Annotation

Sequence reads were dereplicated (100% identity over 100% lengths), then trimmed with the adaptive read trimmer, Sickle (Joshi and Fass, 2011). *De novo* assembly of both metagenomic data sets was done using IDBA UD with a minimum kmer of 52, maximum kmer of 92, and a step size of 8 (Peng et al., 2012). Emergent self-organizing maps of tetranucleotide frequencies were then used to sort scaffolds of length greater than 4,000 bp into genomic bins as described previously (Dick et al., 2009). The Joint Genome Institute Integrated Microbial Genomes and Metagenomics (IMG/M) system was used to annotate genes on assembled scaffolds in the bins of interest (Markowitz et al., 2008). Annotations of genes of interest in these bins were confirmed manually using BLASTp to the National Center for Biotechnology Information non-redundant database with cutoffs of 150 for bitscore and 30% for percent identity (Pruitt et al., 2005), and results were evaluated manually. Scaffold sequences and gene annotations were deposited to NCBI (BioProject PRJNA340051 and PRJNA340052) and Integrated Microbial Genomes (Genome ID 3300003868 and 3300003874), respectively (see **Supplementary Table S1** for accession numbers of individual genome bins).

### Bin Completeness, Taxonomic Identification, and Mapping

Bin completeness was estimated by searching each bin for 36 conserved phylogenetic markers (Ciccarelli et al., 2006). The percentage completeness reported is the number of these conserved phylogenetic markers present out of the expected 36. The taxonomic identification of each bin was determined through a combination of methods including 16S identity, IMG/M, and PhyloSift (Darling et al., 2014). We first conducted a BLASTn search of scaffolds against the Silva Small Subunit RNA database release 119 using cutoffs of 700bp for alignment length and 80% for percent identity (Quast et al., 2013). Abundance of unbinned 16S rRNA genes identified through this BLASTn was then determined through a BLASTn search of all reads against 16S rRNA gene containing portions of scaffolds (Quast et al., 2013). This was then compared to the bin abundances of corresponding taxonomy in order to match unbinned 16S rRNA genes to bins. All bins were further identified using conserved phylogenetic markers as annotated by IMG/M (Ciccarelli et al., 2006). The most resolved taxonomy with ≥50% of gene sequences matching to it was reported (except in the case of Bin A7, in which *Rhodoferax ferrireducens* was reported due to its co-binning with that reference genome). These bin identities were confirmed and resolved using PhyloSift database of 37 universal, conserved gene families (Darling et al., 2014). The percent identity reported for Phylosift analyses refers to the percent of scaffolds that were identified as that taxonomy in the PhyloSift database. Bin abundance was found by mapping all reads to scaffolds of each bin using BWA with default parameters in order to determine coverage (Li and Durbin, 2009). The coverage of each scaffold was normalized for scaffold length and averaged to get mean genome coverage for the bin, which was used as a measure of abundance. In addition, scaffolds from Bin A8 were mapped to a published *Thiothrix lacustris* genome (NCBI accession NZ_JHYQ01000054.1) (Chernousova et al., 2009) using the same method (Li and Durbin, 2009).

## Results and Discussion

**FIGURE 1:**
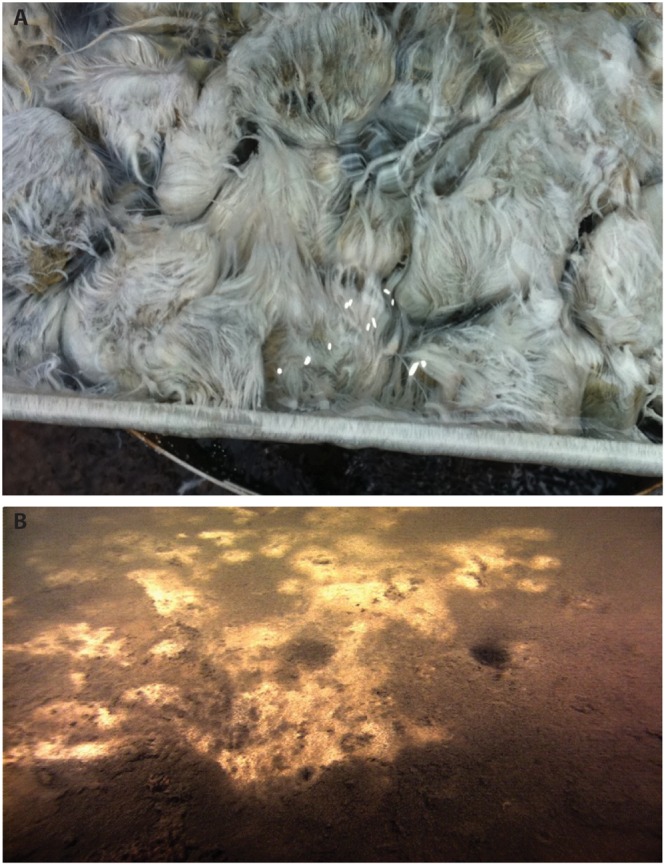
**Images of white microbial mats in the Alpena fountain (A)** and at the Isolated Sinkhole in Lake Huron **(B)**. **(A)** Close-up photograph of *Thiothrix* filaments in the top reservoir of the Alpena fountain. Note that the bright white spots (bottom, center) are an artifact of reflected light in the photograph. **(B)** High-definition video still images of the Isolated Sinkhole taken by the *ROV Little Hercules*, courtesy of Dwight Coleman, Institute for Exploration.

We shotgun sequenced white microbial mat communities that are bathed in similar groundwater at two contrasting locations: the library fountain in the town of Alpena, Michigan (**Figure 1A**; Snider et al., 2017) and the Isolated Sinkhole, which is submerged at a water depth of 93 m in Lake Huron (**Figure 1B**; Biddanda et al., 2006). The emerging groundwater at these two sites is sourced from the same Silurian-Devonian aquifer and has similar conductivity and chemistry (Biddanda et al., 2012). The concentration of H_2_S in water samples taken from the upper basin of the Alpena Fountain, in which the white mats sat, ranged from 17 to 31 μM depending on the exact location and time sampled. Dissolved O_2_ concentrations in the same samples varied from 24 to 28 μM, and the water temperature was 11.4°C. It is unusual to have such relatively high concentrations of H_2_S and dissolved O_2_ in the same water sample. We suspect that this was the result of rapid entrainment of O_2_ from the atmosphere during vigorous mixing that occurs in the fountain. In emerging groundwater of Isolated Sinkhole the dissolved O_2_ concentration ranged from 6 to 44 μM and temperature was 6–7°C.

**FIGURE 2:**
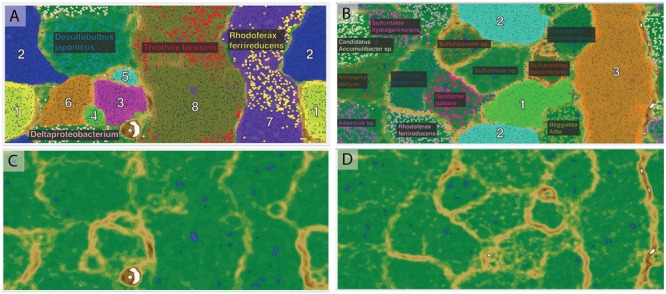
**Emergent self-organizing maps used for binning of scaffolds by tetranucleotide frequency.** The left panels **(A,C)** are for Alpena fountain and the right panels are for Isolated Sinkhole. The top panels **(A,B)** show the assigned bins indicated with numbers and fill color, with small data points representing sequence fragments. The species names indicate publicly available genomes used as references, with their sequence fragments shown in bold. The bottom panels **(C,D)** show the ESOM map background topography, which indicates the difference in tetranucleotide patterns between data points on the map. “High topography” (brown and white) indicates large differences and represents borders between bins, whereas “low topography” (green and blue) indicates similar tetranucleotide frequency patterns and represents areas within genomic bins.

*De novo* assembly and binning yielded eight genomic bins from the Alpena fountain and three from Isolated Sinkhole (**Figure 2**). These genomic bins, which are of varying degrees of completeness, were identified by Phylosift (Darling et al., 2014) and analysis of 16S rRNA genes, and their relative abundance was assessed by mapping reads to binned scaffolds (**Table 1**).

**Table 1:** Properties and taxonomic identification of genomic bins from the Alpena fountain and Isolated Sinkhole.

Bin #	# Scaf	Total length (Mb)	%GC	PhyloSift [% identity]	16S top BLAST hit (% identity), NCBI accession; reference^∗^	Comp	Rel Abund
A1	307	2.0	67.1	*Verrucomicrobia* [50%]	No 16S gene found	75%	1%
A2	486	6.4	61.4	*Desulfobulbaceae* [83%]	*Desulfobulbaceae* clone MS4-14 (99.8%) GQ354922;	64%	2%
A3	209	1.5	31.7	Inconclusive results	*Euryarchaeota* (81.5%) clone 56S_3A_28 DQ837288; López-Archilla et al., 2007.	89%	71%
A4	33	0.2	37.1	*Parcubacteria* [50%]	*Parcubacteria* (86.86%) clone FeSO4_B_5 GQ356988 Beal et al., 2009, unpublished.	78%	1%
A5	27	0.14	40.3	*Elusimicrobia* [87%]	*Elusimicrobia* (97.4%) clone EDW07B003_17, HM066440 Gray and Engel, 2013.	17%	1%
A6	218	1.7	45.4	*Desulfobacteraceae* [100%]	*Desulfobacteraceae* (98.56%) clone 3C9 HQ003582 Ferrer et al., 2011.	61%	1%
A7	44	4.5	58.6	*Rhodoferax* [28%]	*Rhodoferax* sp. (99.9%) clone 015-Cadma AB478656 Horath and Bachofen, 2009, unpublished.	100%	14%
A8	809	10.5	51.3	*Thiothrix* [100%]	*Thiothrix lacustris* (99.0%) EU642572 Chernousova et al., 2009.	89%	10%
IS1	404	2.35	43.0	*Thiotrichaceae* [98%]	*Thiomargarita namibiensis* (89.6%) FR690915 Salman et al., 2011.	39%	40%
IS2	699	4.42	42.4	*Beggiatoa* [68%]	*Beggiatoa* sp. (98.2%) clone: EPS003BBFL_37, JX521065 Headd and Engel, 2014.	89%	47%
IS3	1475	13.48	45.9	*Desulfobacteraceae* [93%]	*Desulfonema* sp. (97.1%) clone: WM28 DQ133916 Macalady et al., 2006.	100%	13%

*#Scaf, number of scaffolds; Comp, completeness; Rel Abund, relative abundance based on read mapping. ^∗^All 16S rRNA genes except the one from IS1 were on scaffolds that were not long enough to be included in the ESOM binning; their assignment is inferred based on similar taxonomy and relative abundance to the rest of the bin.*

### Community Composition

The metagenomes of both mat communities contained abundant members of known groups of LSB. The Isolated Sinkhole metagenome was dominated by two LSB genomic bins. One bin (IS2) represents a near-complete genome that was classified as *Beggiatoa* by both PhyloSift and 16S rRNA gene similarity (**Table 1**). The second (IS1) was classified by Phylosift as family *Thiotrichaceae*, but a fragment (1407 bp) of the 16S rRNA gene sequence had a closest match (89% sequence identity) to *Thiomargarita namibiensis* (Salman et al., 2011), which belongs to one of 8 currently recognized genera within the *Beggiatoaceae.* The 16S rRNA gene sequences associated with these two bins shared 84% sequence identity. The PhyloSift classification uses NCBI taxonomy, based on the system of Garrity et al. (2005), which proposed the *Thiotrichaceae* as a new polyphyletic family that encompasses the three traditional families of LSB, *Leucotrichaceae, Achromatiaceae*, and *Beggiatoaceae*. However, more recent work supports the traditional three family system (Salman et al., 2011, 2013) and thus would consider both bins IS1 and IS2 as members of the *Beggiatoaceae*.

One of the abundant genomic bins in the Alpena fountain (Bin A8) was closely related to *T. lacustris* (**Table 1**), an LSB genus known to form long filaments by attaching to surfaces in habitats with sulfidic flowing water (Larkin and Strohl, 1983), such as the Alpena fountain (**Figure 1**). This identification was supported by PhyloSift classification, by the co-binning of these scaffolds with the *T. lacustris* reference genome (**Figure 2B**), and by mapping of the reads to the *T. lacustris* genome at 157x average coverage. An unbinned 16S gene was found to have a 99% sequence similarity to *T. lacustris* (Chernousova et al., 2009), indicating it is the same species (Yarza et al., 2014).

In addition to LSB, the Isolated Sinkhole and Alpena fountain also had putative sulfate-reducing Deltaproteobacteria. Bins assigned to *Desulfobacteraceae* and *Desulfobulbaceae* were at moderate (Bin IS3; 13%) to low (Bins A2 and A6; 2% and 1%) relative abundances (**Table 1**). Members of these two families are strict anaerobes that reduce sulfate to sulfide (Kuever, 2014a,b). Other members of the microbial mat communities were different between the two sites. The Alpena fountain community is dominated by a well-defined genomic bin (A3; **Figure 2**) whose taxonomic identity could not be resolved by PhyloSift or IMG/M taxonomic profiling. Twenty-six percentage of its gene sequences matched to Archaea and 19% matched to Bacteria. Of the archaeal matches, the most common hits (24%) were to *Euryarchaeota*. An unbinned 16S gene with 97% sequence similarity to an uncultured SM1 euryarchaeon from cold sulfur springs that is highly divergent from cultivated archaea (Rudolph et al., 2001) was found to dominate the 16S rRNA gene sequence reads (**Supplementary Figure S1**) of the fountain, paralleling the dominance of genomic bin A3 of the fountain metagenome (**Table 1**). Members of this lineage, which was recently proposed to be a new euryarchaeal order, *Candidatus* Altiarchaeles (Probst et al., 2014b), form a ‘string of pearls’ morphology and are closely associated with *Thiothrix* filaments (Moissl et al., 2003) by attachment with nano-grappling structures (Moissl et al., 2005; Perras et al., 2014). Given the simplicity of the Alpena fountain community and the parallel dominance of the 16S gene and whole-genome scaffolds, this genomic bin can be confidently assigned as SM1 euryarchaeon.

Also present at high abundance in the Alpena fountain microbial mat was a genomic bin related to *R. ferrireducens* (Bin A7), a facultatively anaerobic betaproteobacterium that oxidizes organic carbon with the reduction of Fe(III) (Finneran et al., 2003). At lower abundance were two unknown groups of bacteria that PhyloSift analyses suggest are *Verrucomicrobia* (Bin1, 50% identity) and *Elusimicrobia* (Bin5, 87% identity) (**Table 1**). Also present at low abundance was a genomic bin for candidate division OD1 (Bin A4) (Harris et al., 2004), which has been found mainly in sulfur-rich, anoxic environments (Peura et al., 2012) and was recently proposed to be named *Parcubacteria* (Rinke et al., 2013).

### Phylogenetic Analysis of LSB

**FIGURE 3:**
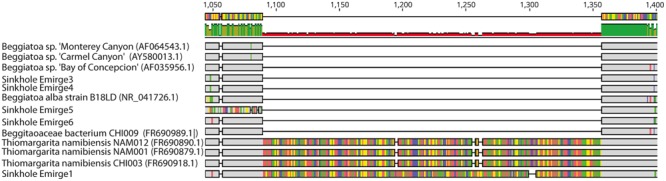
**Geneious screenshot of alignments of 16S rRNA sequences from LSB, showing introns from an Isolated Sinkhole sequence that are homologous to those from *Thiomargarita namibiensis*.** Bases that are dissimilar from other bases in each alignment column are shown in color. Numbers at top indicate the position on the 16S rRNA gene.

To better identify the LSB, phylogenetic analysis was conducted on 16S rRNA and *rpoB* gene sequences assembled from shotgun metagenomic data. Initial alignments revealed the presence of two different large introns in two of the 16S rRNA genes (**Figure 3**). These introns have high sequence similarity to those previously identified in the marine LSB *T. namibiensis* (Salman et al., 2012). Such self-splicing introns are widespread among marine LSB, likely hinder PCR amplification of 16S rRNA genes, and may be horizontally transferred (Salman et al., 2012; MacGregor et al., 2013a; Flood et al., 2016). The introns found here are the first to be identified in non-marine LSB, and given their high sequence similarity to *T. namibiensis*, they are likely the result of horizontal gene transfer. After removal of the introns the remainder of the 16S rRNA gene sequence was most similar to other sequences from Isolated Sinkhole that may represent a novel group in the *Beggiatoaceae* (see below).

**FIGURE 4:**
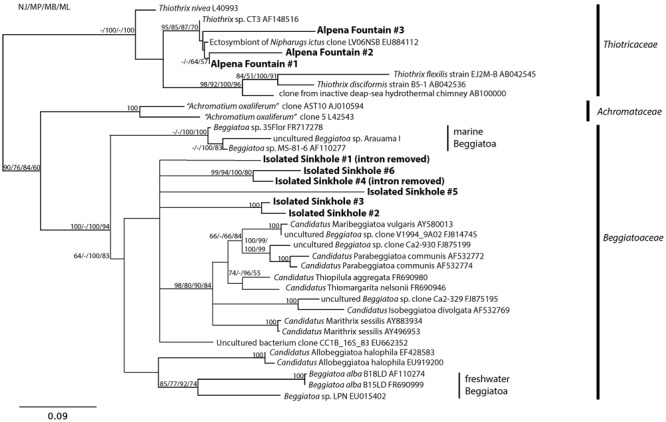
**Phylogenetic tree of the 16S rRNA gene of selected large sulfur bacteria.** Sequences from the Alpena Fountain and Isolated Sinkhole are shown in bold.

Phylogenetic analysis showed that the LSB sequences from Alpena Fountain and Isolated Sinkhole fell within two different clades (**Figure 4**). As expected, the Alpena fountain sequences clustered with *Thiothrix* sequences. The Isolated Sinkhole sequences formed novel clades within the *Beggiatoaceae*. Several branches were unresolved, consistent with previous phylogenetic analyses of these groups (Salman et al., 2013), but results suggest that the Isolated Sinkhole sequences represent new genera that are intermediate between the freshwater and marine *Beggiatoaceae* (**Figure 4**). Similar results were obtained from phylogenetic analysis of *rpoB* gene and protein sequences (**Supplementary Figure S2**). This phylogenetic placement might reflect the salinity of the groundwater fluids at the Alpena fountain and Isolated Sinkhole, which is ∼5% of seawater (Biddanda et al., 2012). However, the absence of such phylogenetically distinct LSB in other environments with comparable salinity, such as the Frasassi and Acquasanta cave systems (Macalady et al., 2008; Jones et al., 2010; Hamilton et al., 2015), suggests that other environmental factors may select for such organisms. Hereafter we refer to these organisms as Isolated Sinkhole *Beggiatoaceae* (ISB).

### Energy Metabolism of LSB

**FIGURE 5:**
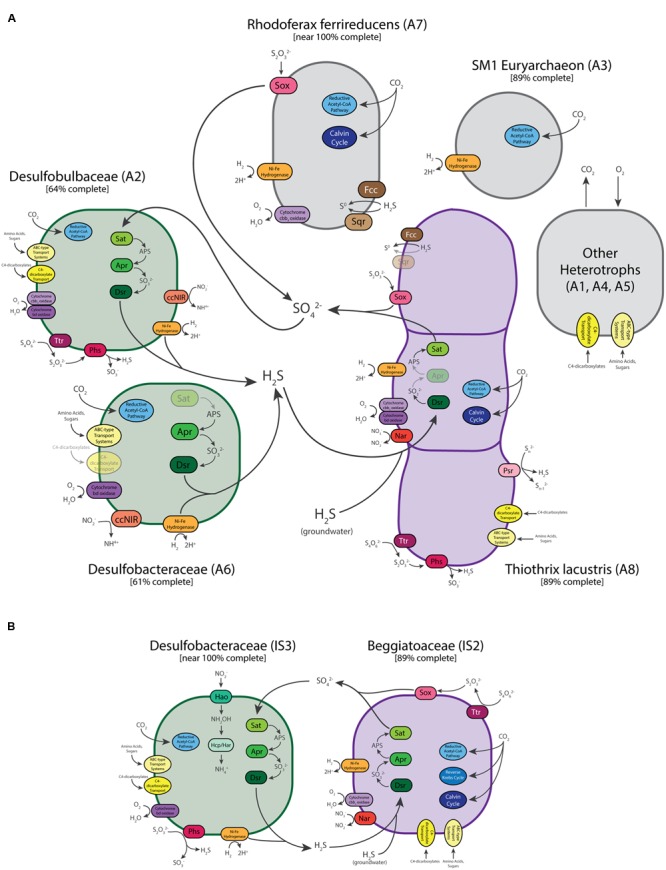
**Schematic illustration of the predicted metabolisms and metabolic interactions of dominant community members of the Alpena fountain (A)** and Isolated Sinkhole **(B)**. Enzymes and reactions for which genes are expected to be present are missing are shown gray/dull. See text for abbreviations.

Genes for dissimilatory sulfur metabolism were prevalent in the metagenomes of microbial mats at both sites. LSB are characterized by their capability to oxidize reduced sulfur compounds for chemolithoautotrophic or mixotrophic growth (Teske and Nelson, 2006). As expected, sulfur oxidation pathways were encoded in the genome bins of *Thiothrix* (A8) and ISB (IS1 and IS2). The *Thiothrix* bin had genes for flavocytochrome c sulfide dehydrogenase (*fcc*), sulfide quinone oxidoreductase (*sqr*), the sox system (*soxABXYZ*) and the reverse dissimilatory sulfite reductase pathway (*dsr*) (**Supplementary Table S2** and **Figure 5**), consistent with the ability of *T. lacustris* to use either sulfide, thiosulfate, or intracellular elemental sulfur globules as electron donors during lithotrophic growth (Chernousova et al., 2009). Bin A8 also contains genes for polysulfide reductase (*psr*), respiratory tetrathionate reductase and thiosulfate reductase (*phs*), which disproportionates thiosulfate into sulfide and sulfite during the respiration of thiosulfate (Akegawa et al., 1985; Stoffels et al., 2012). Thiosulfate reductase genes are also present in *Beggiatoa* genomes (Mußmann et al., 2007), and thiosulfate disproportionation is a key process in some environments with active sulfur cycling (Jørgensen and Bak, 1991). The presence of genes for both oxidation (*sox*) and respiration/disproportionation (*phs*) suggests that it may be a key intermediate in sulfur cycling in the *Thiothrix* mats studied here.

Only one of the two ISB bins from the Isolated Sinkhole (IS2) was near complete (**Table 1**). This genomic bin also contained genes for oxidation of sulfide (*sqr* and *fcc*), thiosulfate (*soxABXY*) and the reverse Dsr pathway (*dsr*, and also *sat* and *apr*) (**Supplementary Table S2** and **Figure 5**), consistent with previously sequenced genomes of the *Beggiatoaceae* (Mußmann et al., 2007; MacGregor et al., 2013b; Flood et al., 2016; Winkel et al., 2016). It also had genes annotated as tetrathionate reductase. The availability of multiple pathways for oxidation of reduced sulfur species in the LSB at both the Alpena fountain and Isolated Sinkhole is consistent with a flexible lifestyle suited to a niche of varying availability of electron donors and acceptors (Klatt and Polerecky, 2015; Winkel et al., 2016).

In addition to lithotrophic growth on reduced sulfur compounds, genes for Ni-Fe hydrogenase in both the *Thiothrix* and ISB bins suggest that these organisms can also use hydrogen as an electron donor. These genes are commonly present in a wide range of organisms and habitats (Greening et al., 2016), including sulfur-oxidizing bacteria (Anantharaman et al., 2013). Members of the *Beggiatoaceae* are known to use H_2_ as a supplemental energy source (Schmidt et al., 1987; Kreutzmann and Schulz-Vogt, 2016; Winkel et al., 2016). H_2_ is produced by fermentation and may be present in terrestrial sulfidic springs (Aragno, 1992).

Both the *Thiothrix* and ISB genomic bins contained evidence of potential for aerobic respiration and nitrate reduction. Two different types of high affinity terminal oxidases for respiration at low-O_2_ concentrations were identified: cytochrome cbb_3_ oxidase and cytochrome bd oxidase (Morris and Schmidt, 2013). The *Thiothrix* bin (A8) contained both of these terminal oxidases, whereas the ISB bin contained only the cbb_3_-type oxidase (**Supplementary Table S2** and **Figure 5**). The presence of these high-affinity terminal oxidases suggest that LSB at each of these sites conduct aerobic respiration at low concentrations of O_2_, which is likely introduced through diffusion and mixing with the atmosphere (Alpena fountain) or with overlying lake water (Isolated Sinkhole). These results are consistent with the microoxic lifestyle of many *Beggiatoa* spp. (Nelson et al., 1986b). To evaluate the possibility that these LSB are also capable of denitrification, we also searched for genes for reduction of nitrate, nitrite, and nitrous and nitric oxides (**Supplementary Table S3**; Philippot, 2002). *Nar* genes for dissimilatory nitrate reduction were found in the LSB genomic bins of both sites (ISB in the Isolated Sinkhole and *T. lacustris* in the fountain), as were *norD* and *norQ* genes for nitric oxide reduction. However, a homolog of the *norB* gene was only found in the *Thiothrix* bin (A8), and genes for dissimilatory reduction of nitrite or nitrous oxide were not found. The pathway for dissimilatory reduction of nitrate to ammonia (nirS and cytochrome c nitrite reductase), which is present in *Thiomargarita* along with genes for classical denitrification to N_2_ (Flood et al., 2016), was also not present in the LSB genomes analyzed here. Thus, we find genomic evidence for only partial denitrification, including reduction of nitrate and potentially nitric oxide. The resulting accumulation of nitrite could necessitate the detoxifying nitrite-reducing enzymes in the sulfate-reducing bacteria (see below). In culture, the type strain for *T. lacustris* is capable of nitrate reduction (Chernousova et al., 2009) but anaerobic growth on nitrate only occurs in the presence of thiosulfate, not organic substrates (Trubitsyn et al., 2013).

### Carbon Metabolism of Large Sulfur Bacteria

Large sulfur bacteria including *Thiothrix* and *Beggiatoa* species have been shown to grow by autotrophy, heterotrophy, and mixotrophy (Nelson and Jannasch, 1983; Nelson et al., 1986a; Schmidt et al., 1987; Chernousova et al., 2009). In low oxygen, sulfidic environments, *Beggiatoa* prefer lithoautotrophic sulfide oxidation (Schmidt et al., 1987). We found genetic evidence of multiple carbon fixation pathways (Berg et al., 2010) in the LSB genomic bins recovered here (**Supplementary Table S2** and **Figure 5**). The *Thiothrix* bin had genetic markers of the Calvin cycle (*rbc* genes) and reductive acetyl-CoA pathway (acyl-CoA synthetase). The reductive acetyl-CoA pathway contains O_2_-sensitive enzymes and is only used by anaerobic or microaerophilic microbes (Hügler and Sievert, 2011). The IS2 ISB bin contained key genes for the 3-hydroxypropionate bicycle (malonyl-CoA reductase, propionyl-CoA synthase and acetyl-CoA carboxylase) and the Calvin cycle (*rbc*). We also found numerous genes annotated as organic carbon transporters in these bins, including transporters for acetate and dicarboxylates. Also present were genes for acetate kinase and acetyl CoA synthetase, which could channel acetate into the general metabolism and are found in *Beggiatoa* genomes (Mußmann et al., 2007; MacGregor et al., 2013b; Flood et al., 2016; Winkel et al., 2016). Genes for oxidation of glycolate, a common photosynthetic exudate, were also found, suggesting an interaction with cyanobacteria (Mußmann et al., 2007). Although no cyanobacteria were detected at Isolated Sinkhole, close relatives of these LSB are tightly associated with cyanobacteria at the Middle Island Sinkhole (Voorhies et al., 2012). Alternatively, glycolate oxidases have been implicated in the consumption of glycolate produced through the oxygenase activity of RubisCO in LSB (Winkel et al., 2016). Many glycolate-related genes were also found in the *Thiothrix* genomic bin (**Supplementary Table S2**). Overall, these results are consistent with the LSB being capable of both autotrophy and heterotrophy, and perhaps mixotrophy.

### Sulfate-Reducing Bacteria

Abundant members of both communities were found to have pathway for dissimilatory sulfate reduction via the *sat, apr*, and *dsr* genes (**Supplementary Table S2**). This suggests that in addition to sulfide present in the groundwater as it emerges from the subsurface, sulfide for the LSB could be sourced from the bacterial reduction of sulfate and/or intermediate sulfur species within the mat (**Figure 5**). The genomic bins that contained genes for sulfate reduction were from *Deltaproteobacteria* groups well known to reduce sulfate. In the Alpena fountain, the genome bin (A2) identified as *Desulfobulbaceae* was the only genomic bin with the entire pathway for reduction of sulfate to sulfide. In addition to producing sulfide via the Dsr pathway, genes for tetrathionate reductase and thiosulfate reductase suggest that sulfide can also be produced via alternative routes involving the reduction and disproportionation of sulfur intermediates (**Figure 5**). It also contained genes for the use of O_2_ as an electron acceptor, H_2_ as an electron donor, a wide variety of organic carbon substrates as a carbon source (**Supplementary Table S2**). This bin also had genes potentially encoding the reductive acetyl-CoA pathway (Wood-Ljungdahl) for carbon fixation (**Supplementary Table S2**). In addition to using this pathway for carbon fixation, it can also be run in reverse and coupled to sulfate reduction to generate energy (Ragsdale and Pierce, 2008). A partial bin of *Desulfobacteraceae* in the Alpena fountain (A6) also had the genes for sulfite and O_2_ reduction, H_2_ oxidation, carbon fixation, and heterotrophy, but it did not have the *sat* or *apr* genes for sulfate reduction. However, a near-complete genomic bin of *Desulfobacteraceae* in the Isolated Sinkhole (IS3) contained the complete pathway along with genes for disproportionation of thiosulfate and the same complement of genes for H_2_ oxidation, O_2_ reduction, carbon fixation, and heterotrophy (**Figure 5**). This bin also contained a 16S rRNA gene sequence that was 97% similar to a sequence from an uncultured deltaproteobacterium of the Frasassi Cave biofilms. This sequence clusters phylogenetically with *Desulfonema* and appears to be filamentous and to have close physical associations with *Beggiatoa* and *Thiothrix* (Macalady et al., 2006). *Desulfobacteraceae* and *Desulfobulbaceae* have also been observed at the Middle Island Sinkhole, in mat consortia of cyanobacteria and LSB (Nold et al., 2010; Kinsman-Costello et al., 2017). Both *Deltaproteobacteria* bins from the Alpena fountain (A2 and A6) had genes for cytochrome c nitrite reductase, which reduces nitrite to ammonium and is used by sulfate-reducing bacteria for detoxification of nitrite, often in association with sulfide-oxidizing, nitrate-reducing bacteria (Haveman et al., 2004). Bin IS3 also had the complete pathway for nitrate reduction to ammonium via the reverse hydroxylamine:ubiquinone reductase module pathway (**Supplementary Table S2**; Hanson et al., 2013).

The genome-predicted phenotypes of H_2_-based autotrophy, organoheterotrophy, respiration of O_2_ have all been observed in cultured sulfate-reducing bacteria including *Desulfobulbaceae* and *Desulfobacteraceae* (Kuever, 2014a,b). Ni-Fe hydrogenase oxidizes H_2_, which is an important electron donor for anaerobic sulfate-reducing bacteria such as the deltaproteobacterium *Desulfovibrio* (Eidsness et al., 1989). Genes for cytochrome oxidases may indicate that the sulfate-reducing bacteria can use oxygen as a terminal electron acceptor for carbon metabolism (Canfield and Des Marais, 1991). Generally this process is linked to ATP production, but not growth (Dilling and Cypionka, 1990; Dannenberg et al., 1992; Sass et al., 2002), although weak growth has been observed (Marschall et al., 1993; Sigalevich and Cohen, 2000).

### SM1 Euryarchaeota

Whereas the Isolated Sinkhole metagenome was dominated by just the two LSB bins and one sulfate-reducing bacterium bin, the Alpena fountain featured additional near-complete genomes from other community members. The genomic bin from the SM1 euryarchaeon accounted for 71% of all mapped metagenomic reads from the fountain (**Table 1**). This lineage is widespread is sulfide springs (Rudolph et al., 2004) but its physiology and potential metabolism have been enigmatic (Probst et al., 2013). Based on close physical associations, syntrophic relationships with *Thiothrix* (Moissl et al., 2002) and sulfate reducing bacteria (Probst et al., 2013, 2014a) have been proposed. Recent genomic data derived from SM1 populations in flowing, oxygen-depleted groundwater in Germany and stratified suboxic/anoxic groundwater in the USA indicate that SM1 can fix carbon via a novel reductive acetyl-CoA (Wood-Ljungdahl) pathway, but no clear genetic evidence of electron donors or acceptors for energy metabolism was uncovered (Probst et al., 2014a). The Alpena fountain SM1 population also has key genes for the reductive acetyl-CoA pathway for carbon fixation, including acetyl-coenzyme A synthetase and CO dehydrogenase/acetyl-CoA synthease (**Supplementary Table S2**). We also found genes for a Ni,Fe-hydrogenase, suggesting an electron donor for lithotrophy, and genes putatively annotated as transporters for amino acids, polysaccharides, and C4-dicarboxylates, suggesting potential for heterotrophy as well (**Figure 5** and **Supplementary Table S2**). Experimental studies are required to confirm these genome-generated hypotheses.

### Other Community Members and Metabolisms

The next most abundant bin in the Alpena fountain was A7, which accounted for 14% of mapped metagenomic reads and was identified as a *Rhodoferax* species based on PhyloSift, 16S rRNA gene sequence, and co-binning with the *R. ferrireducens* genome (**Figure 2**). Consistent with the known metabolic versatility of *R. ferrireducens* (Risso et al., 2009; Yao et al., 2013), we found genes for transporters of organic compounds, sulfide and thiosulfate oxidation, aerobic respiration, dissimilatory nitrate reduction, and autotrophy (**Supplementary Table S2**). Other genomic bins (A1, A4, A5) were present at low relative abundance and the few genes of functional significance noted were related to uptake of organic carbon compounds (**Supplementary Table S2**).

## Conclusion

Metagenomics revealed that the Alpena fountain and Isolated Sinkhole both held relatively low-diversity microbial mat communities supported by sulfur-based chemolithoautotrophy. While taxonomic composition was different, genes underpinning sulfur metabolism, oxygen utilization, carbon fixation, hydrogen oxidation, and denitrification were similar between the two sites. Differences in light availability and water depth do not appear to differentiate the dominant metabolisms of the two communities, but the higher rate of water flow in the Alpena fountain relative to the Isolated Sinkhole is likely a key determinant of which LSB are present. Hence availability of electron donors and acceptors, which is similar between the two sites, likely explains the similar overall metabolisms, while other environmental factors (light, flow) govern differences in richness and phylogenetic diversity. The phylogenetic affinity of Isolated Sinkhole LSB for marine taxa, both in terms of phylogeny and in similarity of introns within the 16S rRNA genes, reveal promising avenues for investigating the biogeography, evolution, and ecology of the *Beggiatoaceae*. Abundant and co-occurring sulfate-reducing organisms alongside the LSB suggest tightly coupled sulfur cycling, echoing microbial metabolic interactions that have been well documented in other environments. Likewise, the discovery that the SM1 euryarchaeon dominates biofilms and co-occurs with *Thiothrix* and sulfate-reducing bacteria in an accessible fountain just outside a city library highlights the potential of this site for studying the biology of this archaeon and its bacterial partners. Overall, this study provides new windows into the diversity and distribution of LSB and their associations with other bacteria as they occur in a lacustrine setting.

## Author Contributions

GD, BB, and AS conceived of this study. GD, DM, BB, and SR collected the samples. AS, BF, JB, DJ, DM, and GD analyzed the data. All authors contributed to writing the manuscript.

## Conflict of Interest Statement

The authors declare that the research was conducted in the absence of any commercial or financial relationships that could be construed as a potential conflict of interest.

## Supplementary Material

FIGURE S1 **Relative abundance of 16S genes as identified by a BLASTn of all reads against portions of scaffolds containing 16S genes found using the Silva119ssu database for (A)** Isolated Sinkhole and **(B)** Alpena fountain communities.

FIGURE S2 **Maximum likelihood phylograms of full-length or nearly full-length (A)** RpoB protein and **(B)** *rpoB* nucleotide sequences. The tree is rooted at the branch separating the *Thiothrix* clade from the other sequences. Numbers indicate bootstrap support for nodes (>50).

TABLE S1 **NCBI and IMG accession numbers for Alpena Fountain and Isolated Sinkhole metagenomes and individual bins**.

TABLE S2 **Annotations and IMG locus tags for key metabolic genes identified across bins**.

TABLE S3 **Key metabolic pathways and genes searched for across bins**.
