# Supplementary material for: Novel Large Sulfur Bacteria in the Metagenomes of Groundwater-Fed Chemosynthetic Microbial Mats in the Lake Huron Basin
Source: Front Microbiol. 2017 May 8;8:791. doi: 10.3389/fmicb.2017.00791 (PMC5421297; doi:10.3389/fmicb.2017.00791)
Supplement: FIGURE S1 — Relative abundance of 16S genes as identified by a BLASTn of all reads against portions of scaffolds containing 16S genes found using the Silva119ssu database for (A) Isolated Sinkhole and (B) Alpena fountain communities. [file Image_1.PDF]

Figure S1:

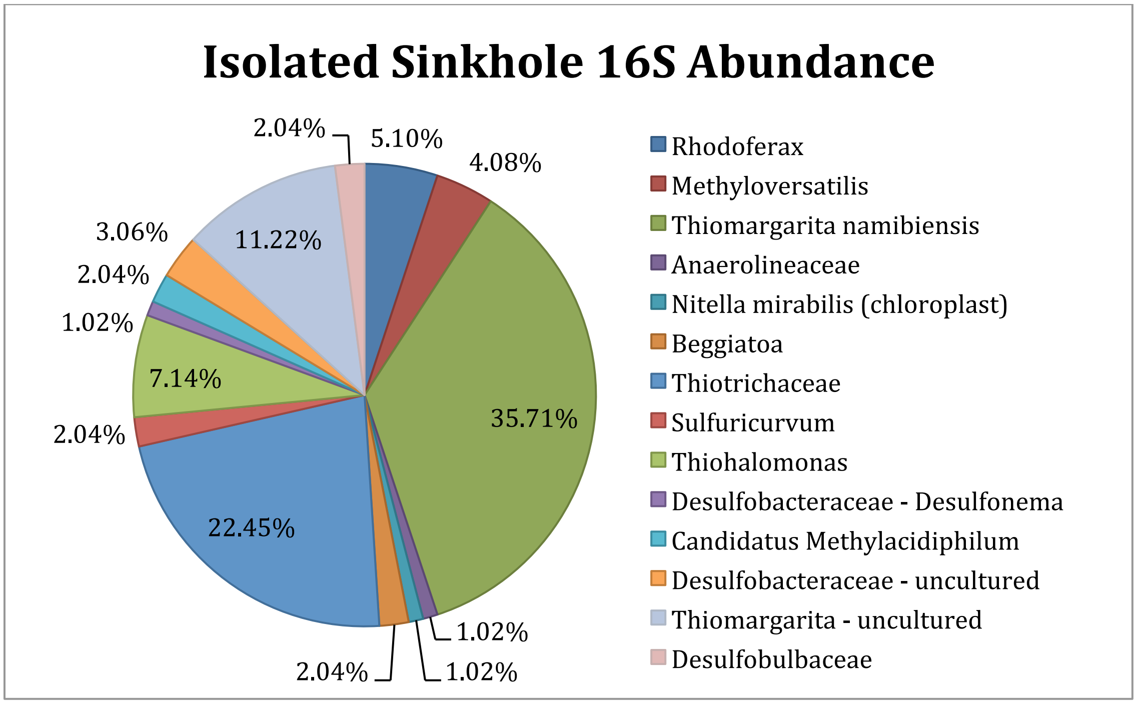

a) Relative abundance of Isolated Sinkhole 16S genes as identified by a BLASTn of all reads against portions of scaffolds containing 16S genes found using the Silva119ssu database.

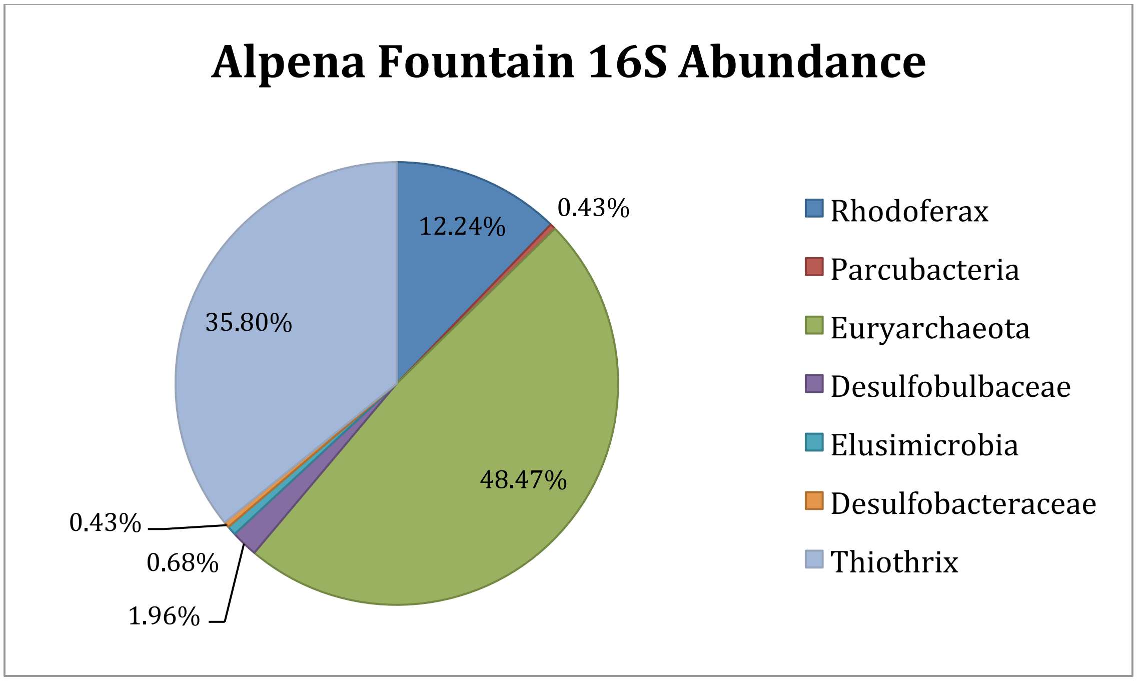

b) Relative abundance of Alpena fountain 16S genes as identified by a BLASTn of all reads against portions of scaffolds containing 16S genes found using the Silva119ssu database.
