# Supplementary figures and images for: Novel Large Sulfur Bacteria in the Metagenomes of Groundwater-Fed Chemosynthetic Microbial Mats in the Lake Huron Basin
Source: Front Microbiol. 2017 May 8;8:791. doi: 10.3389/fmicb.2017.00791 (PMC5421297; doi:10.3389/fmicb.2017.00791)

A

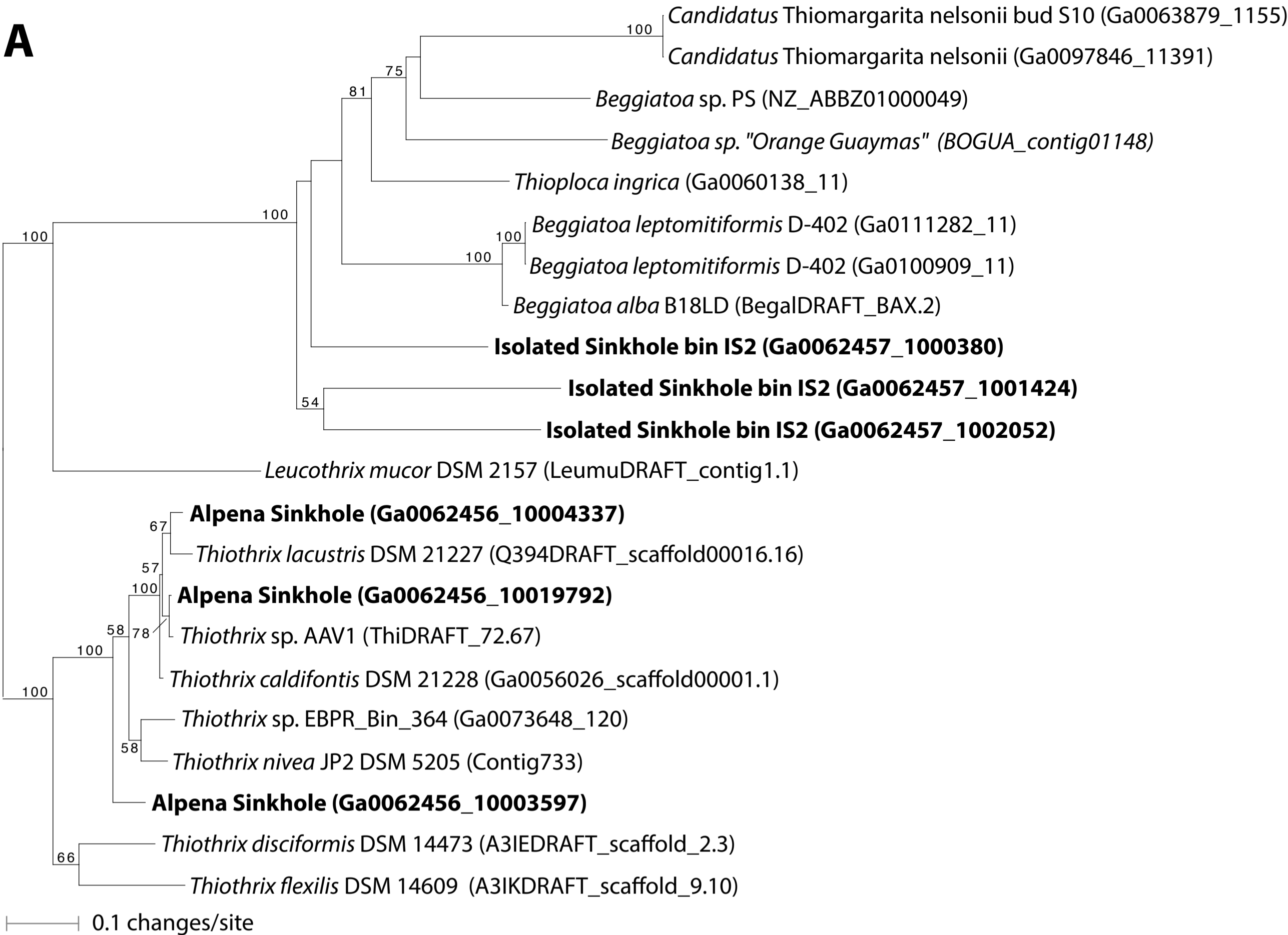

B

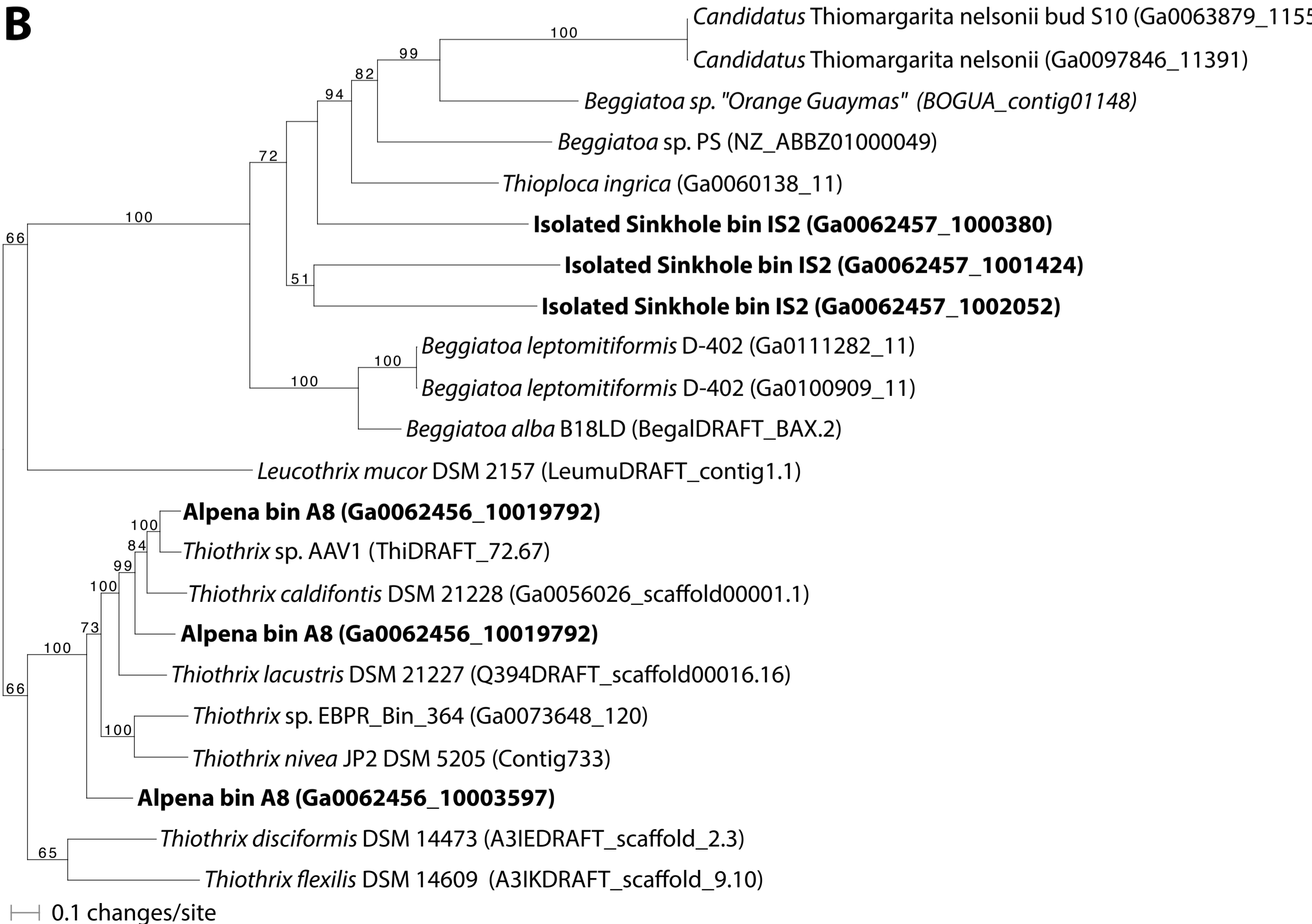

Supplement: FIGURE S2 — Maximum likelihood phylograms of full-length or nearly full-length (A) RpoB protein and (B) rpoB nucleotide sequences. The tree is rooted at the branch separating the Thiothrix clade from the other sequences. Numbers indicate bootstrap support for nodes (>50). [file Image_2.pdf]
